# Cost‐Effectiveness of rTMS as a Next Step in Antidepressant Non‐Responders: A Randomized Comparison With Current Antidepressant Treatment Approaches

**DOI:** 10.1111/acps.13782

**Published:** 2024-12-22

**Authors:** Iris Dalhuisen, Kim Bui, Anne Kleijburg, Iris van Oostrom, Jan Spijker, Eric van Exel, Hans van Mierlo, Dieuwertje de Waardt, Martijn Arns, Indira Tendolkar, Philip van Eijndhoven, Ben Wijnen

**Affiliations:** ^1^ Department of Psychiatry Radboud University Medical Center Nijmegen The Netherlands; ^2^ Donders Institute for Brain Cognition and Behavior Centre for Medical Neuroscience Nijmegen The Netherlands; ^3^ Department of Health Sciences, Faculty of Earth and Life Sciences VU University Amsterdam The Netherlands; ^4^ Center for Economic Evaluation, Trimbos Institute – Netherlands Institute of Mental Health and Addiction Utrecht The Netherlands; ^5^ Department of Health Services Research, CAPHRI Care and Public Health Research Institute Maastricht University Maastricht The Netherlands; ^6^ Neurocare Clinics Nijmegen The Netherlands; ^7^ Depression Expertise Centre, Pro Persona Mental Health Care Nijmegen The Netherlands; ^8^ Behavioral Science Institute Radboud University Nijmegen The Netherlands; ^9^ GGZ inGeest Specialized Mental Health Care Amsterdam The Netherlands; ^10^ Department of Psychiatry Amsterdam University Medical Center Amsterdam The Netherlands; ^11^ Department of Psychiatry & Psychology St. Antonius Hospital Utrecht/Nieuwegein The Netherlands; ^12^ Department of Psychiatry ETZ Hospital (Elisabeth‐TweeSteden Ziekenhuis) Tilburg The Netherlands; ^13^ Research Institute Brainclinics Brainclinics Foundation Nijmegen The Netherlands; ^14^ Faculty of Psychology and Neuroscience Maastricht University Maastricht the Netherlands; ^15^ Stanford Brain Stimulation Lab Stanford University Palo Alto USA

**Keywords:** antidepressants, cost‐effectiveness, major depressive disorder, RCT, rTMS

## Abstract

**Background:**

Although repetitive transcranial magnetic stimulation (rTMS) is an effective and commonly used treatment option for treatment‐resistant depression, its cost‐effectiveness remains much less studied. In particular, the comparative cost‐effectiveness of rTMS and other treatment options, such as antidepressant medication, has not been investigated.

**Methods:**

An economic evaluation with 12 months follow‐up was conducted in the Dutch care setting as part of a pragmatic multicenter randomized controlled trial, in which patients with treatment‐resistant depression were randomized to treatment with rTMS or treatment with the next pharmacological step according to the treatment algorithm. Missing data were handled with single imputations using predictive mean matching (PMM) nested in bootstraps. Incremental cost‐effectiveness and cost‐utility ratios (ICERs/ICURs) were calculated, as well as cost‐effectiveness planes and cost‐effectiveness acceptability curves (CEACs).

**Results:**

Higher QALYs, response, and remission rates were found for lower costs when comparing the rTMS group to the medication group. After 12 months, QALYs were 0.618 in the rTMS group and 0.545 in the medication group. The response was 27.1% and 24.4% and remission was 25.0% and 17.1%, respectively. Incremental costs for rTMS were −€2.280, resulting in a dominant ICUR for QALYs and ICER for response and remission.

**Conclusion:**

rTMS appears to be a cost‐effective treatment option for treatment‐resistant depression when compared to the next pharmacological treatment step. The results support the implementation of rTMS as a step in the treatment algorithm for depression.

**Trial Registration:**

The trial is registered within the Netherlands Trial Register (code: NL7628, date: March 29, 2019)

**Summary**

- Significant Outcomes
  ○ Compared to the next pharmacological treatment step, rTMS showed higher QALYs, response rates, and remission rates, demonstrating superior effectiveness in treatment‐resistant depression.
  ○ rTMS led to lower costs and improved clinical outcomes, with a dominant ICUR and ICER in favor of rTMS over antidepressant medication.
  ○ Over a twelve‐month period, the clinical and economic benefits of rTMS suggest it should be considered a viable option in the treatment algorithm for depression, reinforcing its role in clinical practice.
- Limitations
  ○ Less than half (44.9%) of patients had complete data for all time points, introducing a risk of attrition bias that could compromise the validity and generalizability of the findings.
  ○ Some outcomes were based on self‐report questionnaires, raising the possibility of recall bias and misinterpretation, though the measures used are considered reliable.
  ○ A significant number of patients in the medication group (69%) received rTMS during the follow‐up, which may have blurred group distinctions and underestimated the cost‐effectiveness of rTMS, although it reflects real‐world clinical practice.

## 1 Introduction

Major depressive disorder (MDD) affects nearly 300 million people globally and is associated with chronicity, comorbidity and suicidality, thereby greatly impacting the quality of life [1, 2, 3, 4]. It is a leading cause of years lived with disability (YLD), with an estimated global loss of over 63 million healthy years in 2010 [5]. Besides the substantial burden on patients themselves and those who care for them, the economic impact of depression is considerable. The estimated yearly costs, consisting not only of healthcare costs but also of societal costs such as productivity losses, were estimated at €91 billion and $236 billion in Europe and the U.S. [6, 7].

Despite many effective treatment options, up to 35% of patients do not respond to first‐line treatment such as psychotherapy and antidepressant medication [8]. These patients suffer from treatment‐resistant depression (TRD), which is most commonly operationalized as an inadequate response to at least two treatment trials of adequate dose and duration [9]. Due to the long‐term nature of TRD, as well as the increased risk of suicide, costs are even higher in this population [10, 11]. Considering its high prevalence and substantial ramifications, effective treatment could greatly reduce the social and economic burden of TRD.

Repetitive transcranial magnetic stimulation (rTMS), a non‐invasive brain stimulation modality, is a treatment alternative for depression, with confirmed effectiveness in TRD [12]. In the Netherlands, rTMS combined with psychotherapy has been covered as a treatment for TRD by health care insurance since 2017 [13]. Nonetheless, economic evaluations of rTMS are scarce, especially when comparing rTMS to other treatment options. Previous studies using health economic simulation models have evaluated the cost‐effectiveness of rTMS compared with pharmacotherapy or placebo [14, 15]. Compared to pharmacotherapy, rTMS was associated with an increase in quality‐adjusted life years (QALYs) and a decrease in costs, whereas an increase in both QALYs and costs was observed when compared to placebo. Yet, real‐world evidence of the comparative cost‐effectiveness of rTMS is lacking.

Recently, our group conducted a randomized controlled trial (RCT) in the Netherlands [16, 17], in which the comparative (cost‐)effectiveness of rTMS as an alternative to pharmacotherapy was examined. More specifically, our trial investigated the effectiveness of rTMS in combination with psychotherapy and continued antidepressant medication compared to the next step in the treatment algorithm (i.e., switch or augmentation of antidepressant) in combination with psychotherapy, in a sample of patients with non‐psychotic unipolar depression and moderate treatment resistance. Treatment consisted of 8 weeks, followed by a naturalistic 12‐month follow‐up period. For the clinical effectiveness analysis, we refer to Dalhuisen et al., 2024 [17]. The current paper presents the results of the economic evaluation assessing the cost‐effectiveness of both treatment options, performed alongside the RCT.

### 1.1 Aims of the Study

The aim of this study was to evaluate the cost‐effectiveness of rTMS combined with psychotherapy and continued antidepressant medication compared to the next pharmacological step in the treatment algorithm for patients with TRD. Considering the significant clinical and economic burden of TRD, this study sought to provide real‐world evidence on the comparative long‐term cost‐effectiveness of these two treatment options. Conducted as part of an RCT with a 12‐month follow‐up period, the study examined both QALYs and healthcare costs, aiming to inform healthcare decision‐making regarding the treatment of TRD.

## 2 Materials and Methods

### 2.1 Design

This economic evaluation was conducted from a societal perspective based on data from a pragmatic multicenter RCT. It comprises a cost‐effectiveness and cost‐utility analysis, comparing the costs and effects of the intervention group (rTMS) with those of the control group (medication), with a time horizon of 12 months. In line with the naturalistic follow‐up of the trial, patients could receive care as usual during these 12 months, including rTMS, medication, and other standard treatments for depression. The trial was approved by the Medical Ethics Committee of Arnhem‐Nijmegen (NL68540.091.19) and is registered in the Netherlands Trial Registry (NL7628). Patients gave written informed consent and were explicitly informed before entering the study. Detailed information regarding the methodology and the clinical results of the RCT has been published elsewhere [16, 17].

### 2.2 Clinical Measures

The Dutch Measure for Quantification of Treatment Resistance in Depression (DM‐TRD) was administered at baseline to determine the level of treatment resistance [18]. The HDRS‐17 was used to assess the severity of depressive symptoms at baseline, during treatment, and during follow‐up [19]. Treatment response was defined as a ≥ 50% reduction in score on the HDRS‐17 and remission as a score of < 8. Health‐related quality of life was measured using the EuroQol 5‐dimensions 5‐levels (EQUATION 5D‐5L) [20]. The current health state was assessed across five domains: mobility, self‐care, usual activities, pain, and mood. The health states were expressed as utilities with values ranging from 0 to 1, where 1 corresponds to perfect and 0 to the worst state of health. Utilities were derived using the Dutch Tariffs [21]. QALYs were calculated by using the area under the curve method (i.e., linear interpolation), by multiplying the amount of time a patient spent in a specific health profile with the utility score associated with the health profile [22].

### 2.3 Costs

The economic evaluation was performed according to the Dutch guidelines for economic evaluations [23]. Costs were assessed using the Cost Questionnaire for Psychiatry (Tic‐P) of the Trimbos Institute and Institute for Medical Technology Assessment (iMTA) [24]. A cost‐utility analysis (CUA) with incremental QALYs as outcomes was performed alongside a cost‐effectiveness analysis (CEA) with response and remission rates as outcomes. All costs were calculated using the Dutch standard cost rates (see Supporting Information) [25]. Dutch standard cost rates were indexed to 2022 using the consumer price index of Statistics Netherlands [26]. No discounting was applied since the follow‐up period was exactly 1 year. Four types of costs were included in the analysis: [1] intervention; [2] healthcare utilization; [3] informal care; and [4] productivity losses. Intervention costs consisted of rTMS and psychotherapy sessions. A bottom‐up approach was used to calculate the intervention costs. This was done by multiplying all the treatment steps of each participant by the cost related to the types of treatment. Healthcare utilization costs consisted of consultations with healthcare professionals and medication use. Health care and medication utilization were measured using the self‐reported responses on the Tic‐P questionnaire regarding consultations with health care professionals and medication use during the last 4 weeks. Costs were determined by multiplying health care or medication usage by their corresponding unit prices as published in the Dutch costing manual. Informal care consisted of help from family and friends and transportation. This was assessed based on patients' reports of the number of hours of help they received and multiplying this with the cost price per hour of housekeeping. Productivity costs consisted of losses as a result of absenteeism and presenteeism, both in paid employment and volunteer work. Costs were calculated based on the mean wage per hour and the mean hours a patient normally worked per day. For detailed information on how costs were calculated, see Supporting Information.

### 2.4 Statistical Analysis

All analyses were carried out in R version 4.1.3 following the intention‐to‐treat principle. Missing cost and outcome data were imputed using a single imputation with predictive mean matching (PMM) nested in each bootstrap simulation [27]. PMM was used to account for missing values of the data by imputing real observed values from similar cases. Imputation was based on variables that were associated with missingness and dependent outcomes [28]: gender, randomization group, employment, EQ‐5D‐5L scores at each time point, HDRS scores at each time point and healthcare, informal, and productivity costs at each time point. Subsequently, seemingly unrelated regression equations (SURE) were applied to each bootstrap simulation to account for the correlation between costs and QALYs, response, and remission rates. An advantage of using SURE models is the ability to easily correct for baseline differences of each of the respective outcomes in the regression equations.

The incremental cost‐utility ratio (ICUR) and incremental cost‐effectiveness ratio (ICER) were computed by dividing the incremental costs by incremental effects. This resulted in the costs per QALY, response, and remission rate of the intervention group in comparison to the control group. To quantify the uncertainty around the ICUR/ICER, non‐parametric bootstrapping (5000 times) was performed. This method involves random sampling with replacement from individual data of the patients. Using the results of the non‐parametric bootstrapping procedure, ICURS/ICERs were presented on both cost‐effectiveness planes and cost‐effectiveness acceptability curves (CEACs), in which the probability of the intervention being cost‐effective was shown for a series of willingness‐to‐pay (WTP) thresholds. In the Netherlands, trade‐offs between the burden of the disease and cost‐effectiveness are done in order to assess the maximum incremental costs per QALY; the higher the burden of disease, the higher the willingness to pay for health gains. Since the burden of disease of TRD is 0.64, the Dutch National Health Care Institute proposed a WTP of €50.000 per QALY for TRD interventions [29].

### 2.5 Sensitivity Analyses

To evaluate the robustness of the findings, two one‐way sensitivity analyses were performed. The first sensitivity analysis was conducted from a healthcare perspective, as used for decision‐making in other countries such as the United Kingdom [30]. In this analysis, only intervention and healthcare utilization costs were included (i.e., no productivity losses or patient & family costs). The second sensitivity analysis consisted of using the human capital method, which includes lost productivity costs over the entire period of absence. For this perspective, friction costs were added to the costs considered from the societal perspective.

## 3 Results

### 3.1 Baseline Characteristics

**FIGURE 1:**
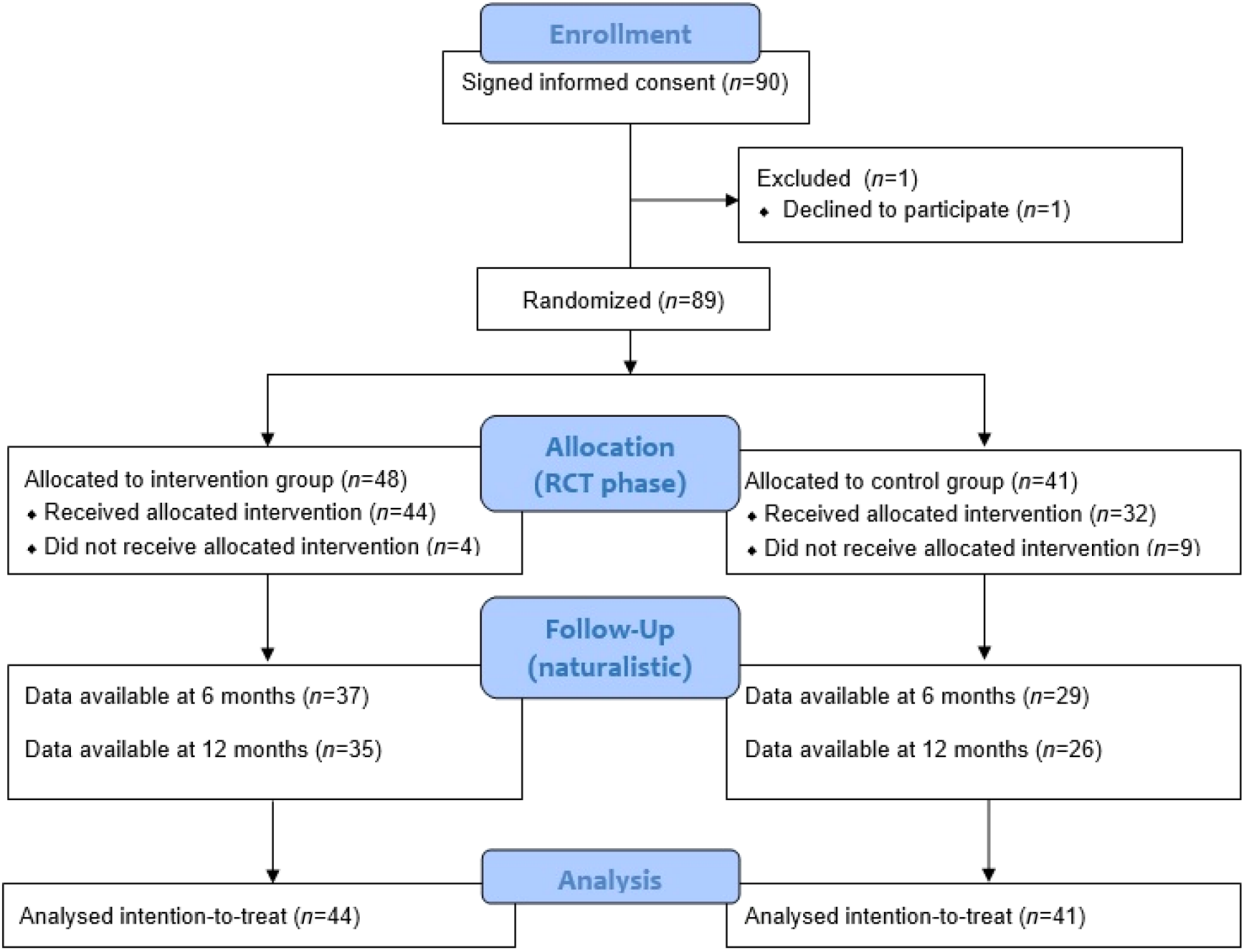
Flowchart.

Ninety patients were included, of which 89 were randomized (see Figure 1 for the CONSORT flowchart). The intervention group consisted of 48 patients, whereas 41 patients were allocated to the control group. Data from six and 12 months follow‐up were available for respectively 37 (77.1%) and 35 (72.9%) patients in the intervention group and 29 (70.7%) and 26 (63.4%) patients in the control group. Baseline characteristics are summarized in Table 1.

**TABLE 1:** Baseline characteristics.

	Total sample (*n* = 89)	Intervention group (*n* = 48)	Control group (*n* = 41)
Gender (female)	59 (66.6%)	31 (64.6%)	28 (68.3%)
Age (years)	43.6 ± 14.5	44.8 ± 14.8	42.3 ± 14.2
DM‐TRD	10.6 ± 2.4	10.7 ± 2.7	10.5 ± 2.1
HDRS‐17	21.4 ± 4.1	21.6 ± 4.1	21.2 ± 4.2
Employed	36 (40.4%)	21 (43.8%)	15 (36.6%)
Healthcare utilization costs	343.1 ± 365.3	366.8 ± 392.1	315.3 ± 333.9
Informal care costs	81.7 ± 315.8	108.0 ± 412.8	50.9 ± 130.7
Costs associated with productivity losses	690.0 ± 1286.1	551.8 ± 992.5	852.4 ± 1560.6
Societal costs	1114.9 ± 1388.5	1026.6 ± 1171.6	1218.6 ± 1616.2

*Note:* Values represent *M* ± SD or *n* (%). Costs are in euros. Abbreviations: DM‐TRD, Dutch measure for treatment resistance in depression; HDRS, Hamilton depression rating scale.

### 3.2 Clinical Effectiveness

The clinical effectiveness after 8 weeks of treatment is discussed elsewhere [17]. An overview of the clinical effectiveness as well as the utilities and QALYs at baseline, six and 12 months can be found in Table 2. HDRS‐17 scores decreased over the 12‐month period in both the intervention and control groups. At 6 months, the mean response rate was 33.3% in the intervention group, and 26.8% in the control group. This decreased slightly at 12 months, to 27.1% and 24.4%, respectively. Remission rates were 22.9% in the intervention group and 22.0% in the control group at 6 months, and 25.0% and 17.1% at 12 months. At baseline, utility scores were slightly higher in the intervention compared to the control group (0.475; 0.462). At 6 and 12 months, a decrease in utility scores was observed in the control group (0.584 and 0.515, respectively). In contrast, in the intervention group, the utility scores increased (0.630 and 0.678, respectively). The intervention group showed higher total QALYs compared to the control group after 6 months (0.271 vs. 0.264). After 12 months both groups showed an increase in QALYs, although this increase was larger in the intervention group (0.076 versus 0.017).

**TABLE 2:** Mean utilities, quality‐adjusted life years (QALYs) and HDRS‐17 scores of the intervention and control groups.

	Intervention group (*n* = 48)	Control group (*n* = 41)
Baseline		
HDRS‐17		
Mean ± SD	21.6 ± 4.1	21.2 ± 4.2
Median [Min, Max]	21.0 [17.0, 34.0]	20.0 [17.0, 39.0]
Missing	0 (0.0%)	0 (0.0%)
Utilities		
Mean ± SD	0.475 ± 0.265	0.463 ± 0.227
Median [Min, Max]	0.474 [−0.237, 0.808]	0.460 [−0.247, 0.844]
Missing	1 (2.1%)	1 (2.4%)
6 months		
HDRS‐17		
Mean ± SD	12.2 ± 7.4	14.6 ± 6.3
Median [Min, Max]	13.0 [0.0, 29.0]	14.5 [3.0, 27.0]
Missing	13 (27.1%)	9 (22.0)
Response	16 (33.3%)	11 (26.8%)
Remission	11 (22.9%)	9 (22.0%)
Utilities		
Mean ± SD	0.630 ± 0.260	0.584 ± 0.290
Median [Min, Max]	0.703 [−0.012, 1.000]	0.655 [0.115, 1.000]
Missing	11 (22.9%)	12 (29.3%)
QALYs^a^		
Mean ± SD	0.271 ± 0.109	0.264 ± 0.107
Median [Min, Max]	0.275 [−0.062, 0.452]	0.272 [−0.091, 0.423]
Missing	11 (22.9%)	12 (29.3%)
12 months		
HDRS‐17		
Mean ± SD	12.1 ± 8.3	13.4 ± 7.6
Median [Min, Max]	12.0 [0.0, 30.0]	12.0 [1.0, 32.0]
Missing	13 (27.1%)	14 (34.1%)
Response	13 (27.1%)	10 (24.4%)
Remission	12 (25.0%)	7 (17.1%)
Utilities		
Mean ± SD	0.678 ± 0.267	0.515 ± 0.290
Median [Min, Max]	0.717 [−0.245, 1.000]	0.658 [−0.016, 1.000]
Missing	13 (27.1%)	15 (36.6%)
QALYs^a^		
Mean ± SD	0.618 ± 0.201	0.545 ± 0.240
Median [Min, Max]	0.635 [0.161, 0.952]	0.595 [−0.122, 0.867]
Missing	13 (27.1%)	16 (39.0%)

^a^ Total QALYs over 6 months; max is 0.5. Values represent *M* ± SD, *median* [min, max] or *n* (%). QALYs: quality‐adjusted life years; HDRS: Hamilton depression rating scale.

### 3.3 Costs

Table 3 shows the mean unadjusted costs per patient. At baseline, the intervention group had higher healthcare and informal care costs compared to the control group. However, throughout the observation period, productivity costs were considerably higher in the control group, resulting in overall higher costs in the control group compared to the intervention group (€1.220 vs. €1.030, respectively). Healthcare utilization, informal care, and productivity costs at 6 months were higher in the control group than in the intervention group. Intervention costs at 6 months were €7.010 in the intervention group and €4.440 in the control group, resulting in higher total costs for the intervention group (€7.670 versus €5.830, respectively). At 12 months, informal costs in the intervention group were slightly higher compared to the control group with a mean difference of €66. Healthcare costs and productivity losses were higher in the control group, resulting in a higher total cost in the control group than in the intervention group (€797 and €609, respectively). In the intervention group, cumulative costs over the full 12‐month period were €3.372 for healthcare utilization, €852 for informal care, and €4.638 for productivity losses. The total cumulative costs were €15.572 in this group. In the control group, these costs were €4.952, €733, and €8.292, respectively, with total cumulative costs of €18.876.

**TABLE 3:** Mean costs (in Euros) and bootstrapped 95% confidence intervals of the intervention and control group at baseline and 6 and 12 months follow‐up over a four‐week period, as well as bootstrapped cumulative costs.

	Baseline^a^		Six months^b^		Twelve months^a^		Cumulative costs	
	Intervention (*n* = 48)	Control (*n* = 41)	Intervention (*n* = 33)	Control (*n* = 26)	Intervention (*n* = 28)	Control (*n* = 18)	Intervention	Control
Intervention								
rTMS	0 **±** 0	0 **±** 0	6210 **±** 130	3600 **±** 3170^c^	0 **±** 0	0 **±** 0		
CBT	0 **±** 0	0 **±** 0	797 **±** 605	680 **±** 412	0 **±** 0	0 **±** 0		
Total (95% CI)^d^	0 (0)	0 (0)	7010 (6240–8163)	4440 (240–8546)	0 (0)	0 (0)	7010 (6240–8163)	4440 (240–8546)
Healthcare utilization								
Primary care^e^	48 **±** 68	63 **±** 109	28 **±** 42	38 **±** 52	32 **±** 53	31 **±** 49		
Mental care^f^	251 **±** 353	208 **±** 240	220 **±** 372	407 **±** 499	120 **±** 189	308 **±** 451		
Other care^g^	32 **±** 88	19 **±** 43	36 **±** 98	10 **±** 37	29 **±** 59	24 **±** 54		
Help at home^h^	25 **±** 72	14 **±** 68	9 **±** 32	22 **±** 72	22 **±** 69	3 **±** 16		
Medication^i^	11 **±** 10	12 **±** 11	8 **±** 9	11 **±** 11	9 **±** 10	14 **±** 11		
Total (95% CI)^d^	367 (0–1344)	315 (0–1023)	301 (0–1282)	488 (0–1834)	211 (0–662)	379 (0–1475)	3372 (2341–4554)	4952 (3532–6548)
Informal care								
Travel	15 **±** 17	13 **±** 14	13 **±** 17	18 **±** 20	9 **±** 11	15 **±** 16		
Help from family/friends	93 **±** 407	38 **±** 128	7 **±** 35	49 **±** 160	119 **±** 436	47 **±** 153		
Total (95% CI)^d^	108 (0–519)	51 (0–566)	20 (0–104)	67 (0–610)	128 (0–1512)	62 (0–561)	852 (286–1823)	733 (269–1454)
Productivity losses								
Paid work, absenteeism	309 **±** 816	442 **±** 1250	95 **±** 306	300 **±** 733	88 **±** 378	206 **±** 591		
Paid work, presenteeism	235 **±** 474	374 **±** 787	208 **±** 522	489 **±** 1140	151 **±** 466	151 **±** 304		
Unpaid work, absenteeism	6 **±** 27	11 **±** 44	5 **±** 22	3 **±** 14	27 **±** 162	0 **±** 0		
Unpaid work, presenteeism	2 **±** 13	26 **±** 87	15 **±** 63	5 **±** 19	4 **±** 20	0 **±** 0		
Total (95% CI)^d^	552 (0–3284)	852 (0–5582)	323 (0–1515)	797 (0–4900)	270 (0–1997)	356 (0–2110)	4638 (2498–7385)	8292 (4574–12,963)
Societal costs								
Total (95% CI)^d^	1030 (0–3806)	1220 (0–5596)	7670 (6314–8921)	5830 (906–10,248)	609 (0–3983)	797 (0–3101)	15,572 (12581–18,562)	18,876 (14437–23,315)
Missing (%)	1 (2.1%)	1 (2.4%)	13 (27.1%)	15 (36.6%)	13 (27.1%)	15 (36.6%)		

*Note:* CI, Confidence Interval. Values represent mean ± SD, unless otherwise indicated.

^a^ Intervention, health care utilization, informal care and productivity costs of the past 4 weeks.

^b^ Intervention costs between baseline and follow‐up at 6 months and health care utilization, informal care, and productivity costs of the past 4 weeks.

^c^ A total of 17 patients of the TAU arm received the intervention.

^d^ Based on 5000 bootstrapped imputations.

^e^ Contact with the general practitioner, or practice nurse.

^f^ Contact with psychiatrist, psychiatric nurse, psychologist, or social worker.

^g^ Contact with physiotherapist, dietician, or alternative healer.

^h^ Home care of family assistance, or other paid help.

^i^ Mood stabilizers, anti‐anxiety, or sleeping pills.

### 3.4 Cost‐Effectiveness

**FIGURE 2:**
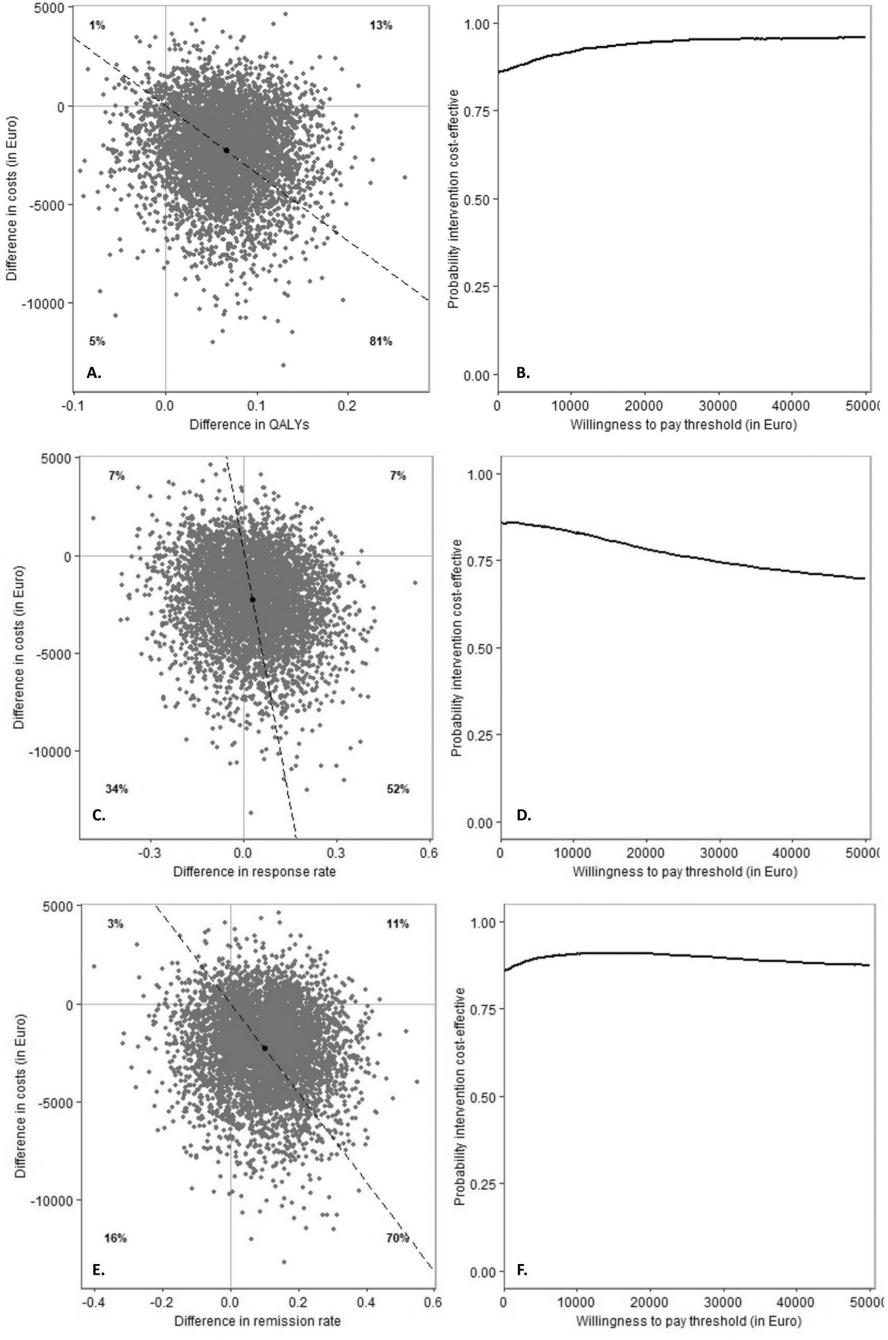
Base case: Societal perspective. (A) Cost‐effectiveness plane of QALY scores at 12 months follow‐up. (B) Cost‐effectiveness acceptability curve of costs per QALY gained at 12 months follow‐up. (C) Cost‐effectiveness plane of response rate at 12 months follow‐up. (D) Cost‐effectiveness acceptability curve of costs per increase of response rate at 12 months follow‐up. (E) Cost‐effectiveness plane of remission rate at 12 months follow‐up. (F) Cost‐effectiveness acceptability curve of costs per increase of remission rate at 12 months follow‐up.

Table 4 presents the adjusted mean costs and effect differences between the intervention and control groups. For QALYs, incremental costs and effects of −€2.280 (95% CI = −€7.165;€1.536) and 0.0664 (95% CI = −0.0231;0.1552) were found, which resulted in a dominant ICUR and indicating that the intervention is both less costly and more effective than the control condition. This is also reflected in the cost‐effectiveness plane, with the majority of the cost‐utility ratios located in the southeast quadrant (see Figure 2A). The CEAC indicates that the probability of the intervention being cost‐effective compared to the control ranges from a minimum of approximately 86% at a WTP of €0 per QALY gained to a maximum of approximately 93% at a WTP of €50.000 (Figure 2B).

**TABLE 4:** Base case and sensitivity analyses based on 5000 bootstrap replications.

	Incremental costs	Incremental effects	Mean ICUR/ICER	Distribution on cost‐effectiveness plane (in %)			
				NE	SE	SW	NW
Cost‐utility analysis – QALYs							
Societal perspective	−€2.280 (−€7.165; €1.536)	0.0664 (−0.0231; 0.1552)	Dominant (lower costs, higher effects)	13	81	5	1
Health care perspective	−€1.313 (−€3.195; €446)	0.0664 (−0.0231; 0.1552)	Dominant (lower costs, higher effects)	6	88	5	1
Human capital approach	−€2.425 (−€7.206; €1.409)	0.0664 (−0.0231; 0.1552)	Dominant (lower costs, higher effects)	11	83	5	1
Cost‐effectiveness analysis—response rate							
Societal perspective	−€2.280 (−€7.164; €1.536)	0.0263 (−0.2249 0.2733)	Dominant (lower costs, higher effects)	7	52	34	7
Health care perspective	−€1.313 (−€3.195; €446)	0.0263 (−0.2245 0.2733)	Dominant (lower costs, higher effects)	3	56	37	4
Human capital approach	−€2.425 (−€7.206; €1.409)	0.0263 (−0.2249 0.2733)	Dominant (lower costs, higher effects)	6	53	35	6
Cost‐effectiveness analysis – remission rate							
Societal perspective	−€2.280 (−€7.165; €1.536)	0.1003 (−0.1273 0.3259)	Dominant (lower costs, higher effects)	11	70	16	3
Health care perspective	−€1.312 (−€3.195; €446)	0.1003 (−0.1273 0.3259)	Dominant (lower costs, higher effects)	5	76	17	2
Human capital approach	−€2.425 (−€7.206; €1.409)	0.1003 (−0.1273 0.3259)	Dominant (lower costs, higher effects)	9	72	16	3

*Note:* Values represent mean (95% CI) or %. Abbreviations: ICER, incremental cost‐effectiveness ratio; ICUR, incremental cost‐utility ratio; NE, north‐east; NW, north‐west; SE, south‐east; SW, south‐west.

For response rate, incremental costs and effects of −€2.280 (95% CI = −€7.165; €1.536) and 0.0263 (95% CI = −0.2249; 0.2733) were found, resulting in a dominant ICER. Cost‐effect ratios were mostly located in the southeast quadrant of the cost‐effectiveness plane (Figure 2C), in which higher response rates are found for lower costs in the intervention group. The CEAC shows that the probability of the intervention being cost‐effective compared to the control ranges from a minimum of 75% at a WTP of €50.000 per response gained to a maximum of approximately 86% at a WTP of €0 (Figure 2D).

For the remission rate, incremental costs and effects of −€2.280 (95% CI = −€7.165; €1.536) and 0.1003 (95% CI = −0.1273; 0.3259) also led to a dominant ICER. The cost‐effect ratios primarily fell within the southeast quadrant of the cost‐effectiveness plane (Figure 2E), indicating higher remission rates at lower costs in the intervention group compared to the control group. The CEAC implies that the probability of the intervention being cost‐effective compared to TAU ranged from a minimum of approximately 86% at a WTP of €0 per remission gained to a maximum of approximately 90% at a WTP of €15.000 (Figure 2F).

### 3.5 Sensitivity Analyses

By applying the healthcare perspective, the cost difference between the intervention and control group decreased from −€2.280 to −€1.313 (see Table 4). The intervention remains the dominant choice, with a greater percentage of mean ICERs/ICURs in the southeast quadrant when compared to the base case (see Supporting Information). When applying the human capital approach to calculate productivity losses, an increase in mean productivity costs (€16.932) in comparison to the friction cost method (€16.797) was observed. As a result, incremental costs of −€2.425 were found, demonstrating a slight improvement in cost‐effectiveness for the intervention (see Supporting Information).

## 4 Discussion

This study examined the cost‐effectiveness of rTMS versus antidepressant medication across different outcomes for patients with moderate treatment‐resistant depression [31]. Despite intervention costs being higher for rTMS, higher QALYs, response, and remission rates were found for lower costs when comparing rTMS to antidepressants, resulting in cost‐utility and cost‐effectiveness ratios located primarily in the south‐east quadrant, indicating a dominant ICER and hence rTMS being the cost‐effective treatment. At a WTP threshold of €50.000 per QALY gained, the Dutch reference for TRD interventions, the probability of rTMS being cost‐effective was 93% [29]. For response and remission, the optimal values were 86% at a WTP of €0 and 90% at €15.000. In contrast to the cost per QALY found in CUAs, no reference WTP thresholds exist for CEAs, which assign a monetary value to, for example, a certain improvement in response or remission. Looking at a proxy for the WTP for improvement in response and remission, a recent study reported mean total costs of €21,186 per depressive episode, which is substantially higher than our reported values [32]. Moreover, given that rTMS resulted in higher effects and lower costs, one might argue that a WTP value is redundant in this case. The results of our sensitivity analyses are in line with the main analysis, indicating rTMS as the dominant intervention.

Although no comparative cost‐effectiveness analyses of rTMS and antidepressant medication have been performed based on real data, several simulations have been performed. A study based on the Japanese setting showed that rTMS was more cost‐effective than medication in TRD patients, with an ICER of $6832 per QALY gained [33]. Furthermore, a scenario analysis indicated that this was also the case for the reintroduction of rTMS upon relapse. In a Markov model based on the Hungarian healthcare context, rTMS was compared to standard third‐line treatment, which could include an antidepressant switch, augmentation, or ECT [34]. The ICER was €14,670 per QALY gained, in favor of rTMS. Whereas the previous study was based on a time horizon of a year, Voigt and colleagues performed a lifetime simulation of depressed patients who had failed to benefit from one pharmacotherapy trial [35]. Their results indicated rTMS to be dominant, even after a single failed antidepressant treatment trial. This finding aligns with the (clinical) results of our trial, supporting the earlier application of rTMS, rather than after multiple failed pharmacotherapy trials.

The substantial cost‐effectiveness of rTMS despite its higher costs may be explained by several factors. Firstly, rTMS led to lower observed costs in healthcare utilization and productivity losses, which could be explained by its higher clinical effectiveness, as increased mental well‐being is associated with lower healthcare costs as well as lower amounts of sick leave [36]. In contrast, informal care costs were higher in the rTMS group, which could be explained by an increase in emotional and practical support from family and friends after the reduction of professional healthcare services. Nevertheless, the savings in healthcare utilization and productivity losses outweigh the higher informal care costs in the rTMS group. Second, the dominant cost‐effectiveness of rTMS could also result from the large difference in clinical effectiveness between the rTMS and medication groups. Although direct comparisons between rTMS and antidepressant medication are scarce, the effectiveness of antidepressants in patients with TRD is generally considered to be low [8]. In comparison, ECT is considered an effective treatment option, even in TRD, although it is also more invasive. Cost‐effectiveness analyses comparing rTMS and ECT mostly show that ECT is the more cost‐effective option [37, 38]. However, a lifetime simulation using a Markov model showed that rTMS is dominant over ECT from the societal perspective, although the highest QALY gains and greatest cost savings were achieved when rTMS non‐responders switched to ECT [39].

### 4.1 Strengths and Limitations

This study has several strengths. First, when designing the RCT the Dutch guidelines for economic evaluations were taken into consideration, ensuring that best practices in economic evaluation were incorporated. Second, as the data come from an RCT with a follow‐up period of 12 months, data collection is extensive. This allows for a detailed analysis of the (long‐term) outcomes and impacts and reduces reliance on assumptions based solely on literature. Third, the multiple sensitivity analyses allow for a comprehensive exploration of the robustness and reliability of the results. However, some limitations also exist. First, less than half of the patients (44.9%) had complete data for all time points. Since the majority of the sample had incomplete data, attrition bias might have occurred, potentially compromising the validity and generalizability of the results [40]. Second, outcomes were partly based on self‐report measures which could introduce recall bias and misinterpretation. However, these questionnaires have been proven to be reliable and feasible [41, 42]. Finally, the follow‐up of this RCT was naturalistic, meaning that all patients could receive treatment with rTMS, medication, or another intervention after the initial eight‐week treatment phase. While this approach was ethically necessary to ensure that all patients had access to all potentially beneficial treatments, it introduces the possibility of biased results. As a substantial number of patients (69%) in the medication group received rTMS at some point during the follow‐up period, this cross‐over blurred the distinction between the rTMS and medication groups, potentially underestimating the cost‐effectiveness of rTMS. Nevertheless, this situation reflects real‐life clinical practice and, as such, provides valuable information on the cost‐effectiveness of interventions for patients with TRD.

### 4.2 Future Directions

Future research on the cost‐effectiveness of this rapidly evolving treatment should take new developments into account. The rTMS protocol of the current study consisted of 25 sessions, whereas recent research has shown that effectiveness is greatest after at least 30 sessions [43]. As the direct costs associated with rTMS are relatively high, this increase in the number of sessions could influence the cost‐effectiveness of rTMS. Nevertheless, in the study by Voigt and colleagues, rTMS was still the dominant treatment in a sensitivity analysis examining a protocol of 28–34 treatment sessions [35]. Another development pertains to intermittent theta‐burst stimulation (iTBS). In this specific treatment protocol, stimulation is applied in bursts of 50 Hz, resulting in a much shorter duration time of only 3 min compared to the 18–30 min of a ‘regular’ rTMS protocol. As the duration of a session is considerably shorter, the costs associated with the treatment are also lower. Indeed, a cost analysis comparing iTBS and conventional rTMS showed substantial cost savings both per patient and per remission [44]. Finally, as the likelihood of achieving remission with medication decreases with each successive treatment attempt, the cost‐effectiveness of applying rTMS at an earlier stage, e.g., in medication‐naïve patients or after a first failed treatment, should be investigated. Based on a lifetime Markov simulation, rTMS was the dominant therapy compared to antidepressant medication in patients with newly diagnosed depression who had failed one treatment trial [35].

### 4.3 Conclusion

In the Dutch setting, rTMS appears to be a cost‐effective treatment alternative compared to the next pharmacological step in the treatment algorithm for patients with a moderate level of treatment‐resistant depression. Despite the higher costs of rTMS compared to medication, the incremental costs over the long term are lower. The results from this study support the implementation of rTMS in the treatment algorithm and are valuable not only for national policymakers but also at the international level, as they contribute to informed decision‐making on how to allocate limited resources effectively.

## Author Contributions

P.v.E., I.T., J.S. designed the current trial, with PvE as the principle investigator. The manuscript draft was written by I.D., K.B., P.v.E., B.W., I.D., K.B. performed the data analysis. All authors contributed to the interpretation of the data. All authors gave valuable feedback on the manuscript and approved the final version.

## Ethics Statement

The trial was approved by the Medical Ethics Committee of Arnhem‐Nijmegen (NL68540.091.19) and is registered with the Netherlands Trial Registry (NL7628). All participants gave written informed consent according to the Declaration of Helsinki before entering the study and were explicitly informed that they could withdraw from participation at any time without any further explanation.

## Consent

The authors have nothing to report.

## Conflicts of Interest

The authors declare no conflicts of interest.

### Peer Review

The peer review history for this article is available at https://www.webofscience.com/api/gateway/wos/peer‐review/10.1111/acps.13782.

## Supporting information


Data S1.

## Data Availability

The datasets that were used during the current study are available in the Radboud Repository, https://repository.ubn.ru.nl/.
