# Supplementary material for: Cost‐Effectiveness of rTMS as a Next Step in Antidepressant Non‐Responders: A Randomized Comparison With Current Antidepressant Treatment Approaches
Source: Acta Psychiatr Scand. 2024 Dec 22;151(5):613–24. doi: 10.1111/acps.13782 (PMC11962342; doi:10.1111/acps.13782)
Supplement: Supplementary file 1 — Data S1. [file ACPS-151-613-s001.docx]

**Cost-effectiveness of rTMS as a next step in antidepressant non-responders: a randomized comparison with current antidepressant treatment approaches**

Iris Dalhuisen, Kim Bui, Anne Kleijburg, Iris van Oostrom, Jan Spijker, Eric van Exel, Hans van Mierlo, Dieuwertje de Waardt, Martijn Arns, Indira Tendolkar, Philip van Eijndhoven, & Ben Wijnen

*Supplementary materials*

*Patients*

Adult patients were recruited from outpatient clinics across the Netherlands. Inclusion criteria were moderate to severe (Hamilton Depression Rating Scale (HDRS-17) score > 16) unipolar MDD without psychotic symptoms (Structured Clinical Interview for DSM-5 disorders (SCID-I; 2018), with inadequate response to at least two treatment trials (of which at least one antidepressant trial of adequate dose and duration) and a current depressive episode duration of less than two years. rTMS contraindications were the only exclusion criteria (see accompanying papers for a full list). If patients were taking antidepressant medication, this needed to be stable for a minimum of six weeks.

*Procedure*

Patients were recruited between August 2019 and December 2022. After giving informed consent, patients were randomized to either the intervention or control group. Data for the economic evaluation was collected via clinical interviews and self-report questionnaires at baseline, and six and twelve months follow-up.

*Interventions*

rTMS – Treatment with rTMS consisted of a 10 Hz protocol, applying 3000 pulses per session at an intensity of 120% of the resting motor threshold (rMT) and targeting the left dorsolateral prefrontal cortex (DLPFC). Over the course of eight weeks, twenty five sessions were scheduled.

Medication - Treatment as usual with medication consisted of protocolled pharmacological treatment steps that are prescribed within the Dutch guideline, e.g. a switch from the current antidepressant medication to a tricyclic antidepressant (TCA) or augmentation of the current antidepressant medication with lithium or a second-generation antipsychotic (18). If this was not a suitable option, e.g. for medical reasons, a different antidepressant could be prescribed, as would be the case during usual care.

Psychotherapy – in both groups patients received psychotherapy sessions either in a group or individually, at least once a week. This could consist of cognitive behavioral therapy, behavioral activation therapy, or supportive therapy, and differed between patients and treatment centers, as it would during usual care.

*Costs*

The economic evaluation was performed according to the Dutch guidelines for economic evaluations (24). A cost-utility analysis (CUA) with incremental QALYs as outcomes was performed alongside a cost-effectiveness analysis (CEA) with response and remission rates as outcomes. All costs were calculated using the Dutch standard cost rates (see supplementary materials) (25). Dutch standard cost rates were indexed to 2022 using the consumer price index of Statistics Netherlands (26). No discounting was applied, since the follow-up period was exactly one year. Four types of costs were included in the analysis: (1) intervention; (2) healthcare utilization; (3) informal care; and (4) productivity losses. For detailed information on how costs were calculated, see supplementary materials.

Intervention costs consisted of rTMS and psychotherapy sessions. A bottom-up approach was used to calculate the intervention costs. This was done by multiplying all the treatment steps of each participant by the cost related to the types of treatment.

Healthcare utilization costs consisted of consultations with healthcare professionals and medication use. Health care and medication utilization were measured using the report of the patients regarding their consultations with health care professionals and their medication use during the last four weeks. The healthcare utilization costs were calculated by multiplying the healthcare utilization with the price of the corresponding consultation (25). Medication costs were calculated by multiplying the medication utilization with the medication cost price, using prices based on Daily Defined Dosage (DDD) taken from www.farmacotherapeutischkompas.nl and www.medicijnkosten.nl.

Informal care consisted of help from family and friends and transportation. Help from family and friends was measured using the report of the patients regarding the number of hours they received help from family and friends in their housekeeping in the past four weeks. The costs associated with received help were calculated by multiplying the total hours with the cost price per hour for housekeeping. Transportation was measured using the report of the patients regarding the number of consultations with healthcare professionals during the last four weeks. Transportation costs were calculated by multiplying this number of consultations with the average distance to each location (including parking fees) and the costs per kilometer.

Productivity costs consisted of losses as a result of absenteeism and presenteeism, both in paid employment and volunteer work. Absenteeism costs in paid employment were calculated following the friction cost method, in which the assumption is made that absent employers will be replaced after a specified friction period. The Netherlands calculated a friction period of 85 business days for 2014, corresponding to 136 business days in 2022 (25). Absenteeism was measured using the report of the patients regarding their number of absent days during the last four weeks. Absenteeism costs were calculated by multiplying the number of absent days by the mean wage per hour and the mean hours the patient normally worked per day. Presenteeism was measured using the report of the patients on their productivity at work while experiencing health issues. Presenteeism costs were calculated by multiplying the number of present days by the mean wage per hour, the mean hours worked per day, and their productivity level in percentages.

*Prices per category in Euros*

| **Category** | **Price (€)** |
| --- | --- |
| **Intervention (per session)** | |
| rTMS | 249.58 |
| CBT | 120.20 |
| **Healthcare utilization (per consult)** | |
| General Practitioner | 40.31 |
| Practice nurse | 20.77 |
| Psychiatric nurse | 85.34 |
| Social worker | 79.41 |
| Physiotherapist | 40.31 |
| Dietitian | 40.31 |
| Psychologist | 78.18 |
| Psychiatrist | 136.82 |
| Alternative practitioner | 104.48 |
| Home care/family support | 20.00 |
| Help at home and other | 26.27 |
| **Medication (per day)** | |
| Antidepressants | 0.48 |
| Anxiety/tension/nervousness | 0.39 |
| Hypnotics | 0.10 |
| **Transport (per visit)** | |
| Parking ticket | 3.00 |
| General Practitioner | 0.12 |
| Practice nurse | 0.23 |
| Psychiatric nurse | 0.23 |
| Social worker | 0.12 |
| Physiotherapist | 0.23 |
| Dietitian | 0.23 |
| Psychologist | 0.39 |
| Psychiatrist | 0.23 |
| Alternative practitioner | 1.33 |
| **Help from family/friends (per hour)** | |
| Family/friends/neighbours/housekeeping | 17.10 |
| Unpaid help and other | 14.00 |
| **Productivity losses (per hour)** | |
| Paid work | 37.11 |
| Unpaid work | 14.95 |

*Sensitivity analysis: Healthcare perspective*

| 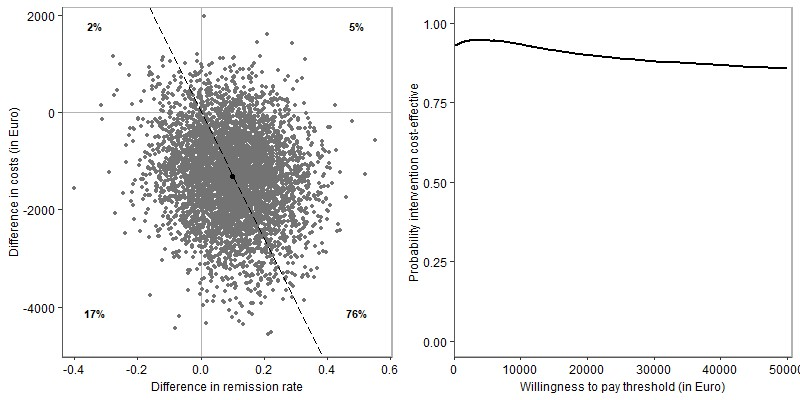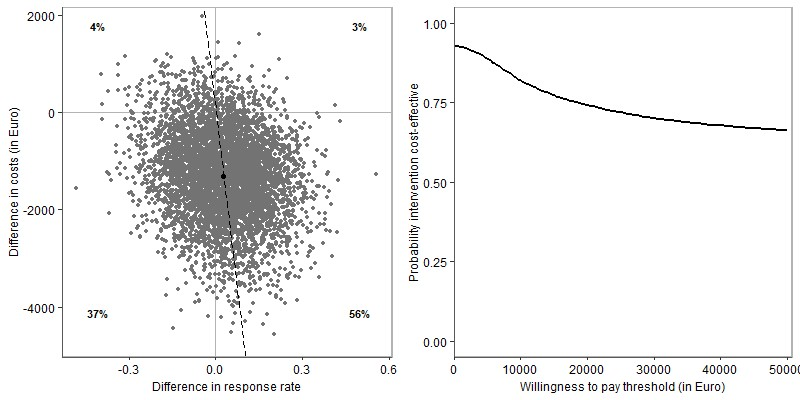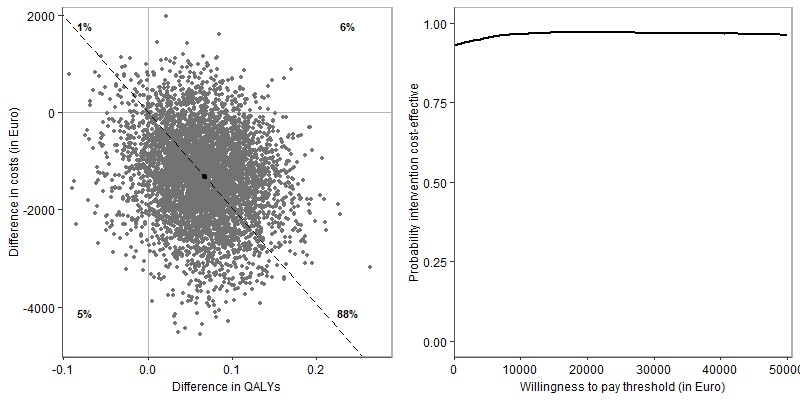  **D.**  **C.**  **B.**  **A.** |
| --- |
| **A).** Cost-effectiveness plane of QALY scores at 12 months follow-up. **B).** Cost-effectiveness acceptability curve of costs per QALY gained at 12 months follow-up. **C).** Cost-effectiveness plane of response rate at 12 months follow-up. **D).** Cost-effectiveness acceptability curve of costs per increase of response rate at 12 months follow-up. **E).** Cost-effectiveness plane of remission rate at 12 months follow-up. **F).** Cost-effectiveness acceptability curve of costs per increase of remission rate at 12 months follow-up.  **E.**  **F.** |

*Sensitivity analysis: Human capital approach*

| 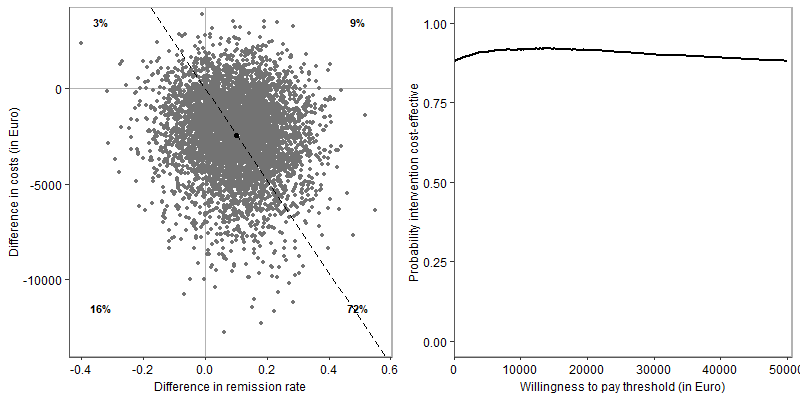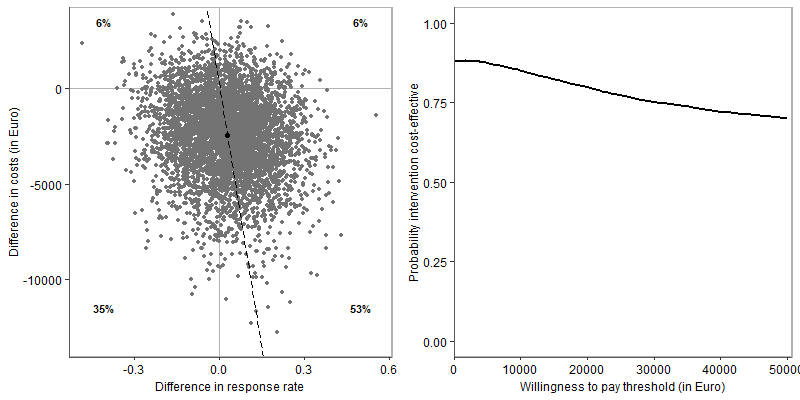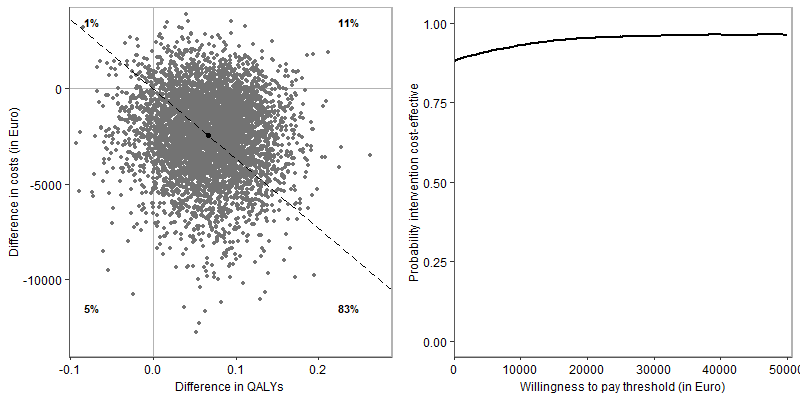  **D.**  **C.**  **B.**  **A.** |
| --- |
| **A).** Cost-effectiveness plane of QALY scores at 12 months follow-up. **B).** Cost-effectiveness acceptability curve of costs per QALY gained at 12 months follow-up. **C).** Cost-effectiveness plane of response rate at 12 months follow-up. **D).** Cost-effectiveness acceptability curve of costs per increase of response rate at 12 months follow-up. **E).** Cost-effectiveness plane of remission rate at 12 months follow-up. **F).** Cost-effectiveness acceptability curve of costs per increase of remission rate at 12 months follow-up.  **F.**  **E.** |
